# Supplementary material for: Prevalence of intestinal protozoan parasites among school children in africa: A systematic review and meta-analysis
Source: PLoS Negl Trop Dis. 2022 Feb 11;16(2):e0009971. doi: 10.1371/journal.pntd.0009971 (PMC8870593; doi:10.1371/journal.pntd.0009971)
Supplement: S1 Table — (DOCX) [file pntd.0009971.s002.docx]

| **S1 Table.** Search strategies | |
| --- | --- |
| **Databases** | **Search strategies** |
| **PubMed** | (((((((((((((((((((((((“intestinal parasites”[Title/Abstract]) OR “intestinal parasite”[Title/Abstract]) OR “intestinal parasitic”[Title/Abstract]) OR “intestinal protozoa”[Title/Abstract]) OR “protozoan parasites”[Title/Abstract]) OR “gastrointestinal protozoa”[Title/Abstract]) OR “parasitic diarrhoea”[Title/Abstract]) OR “parasitic diarrhea”[Title/Abstract]) OR entamoeba[Title/Abstract]) OR giardia[Title/Abstract]) OR cryptosporidium[Title/Abstract]) OR balantidium[Title/Abstract]) OR cyclospora[Title/Abstract]) OR cystoisospora[Title/Abstract]) OR amebiasis[Title/Abstract]) OR amoebiasis[Title/Abstract]) OR giardiasis[Title/Abstract]) OR cryptosporidiosis[Title/Abstract]) OR balantidiasis[Title/Abstract]) OR cyclosporiasis[Title/Abstract]) OR cystoisosporiasis[Title/Abstract])) AND (((children[Title/Abstract]) OR paediatric[Title/Abstract]) OR pediatric[Title/Abstract])) AND ((((((((((((((((((((((((((((((((((((((((((((((((((((((Algeria[Title/Abstract]) OR Egypt[Title/Abstract]) OR Libya[Title/Abstract]) OR Morocco[Title/Abstract]) OR “South Sudan”[Title/Abstract]) OR Sudan[Title/Abstract]) OR Tunisia[Title/Abstract]) OR Burundi[Title/Abstract]) OR Comoros[Title/Abstract]) OR Djibouti[Title/Abstract]) OR Eritrea[Title/Abstract]) OR Ethiopia[Title/Abstract]) OR Kenya[Title/Abstract]) OR Madagascar[Title/Abstract]) OR Malawi[Title/Abstract]) OR Mauritius[Title/Abstract]) OR Mozambique[Title/Abstract]) OR Rwanda[Title/Abstract]) OR Seychelles[Title/Abstract]) OR Somalia[Title/Abstract]) OR Tanzania[Title/Abstract]) OR Uganda[Title/Abstract]) OR Zambia[Title/Abstract]) OR Zimbabwe[Title/Abstract]) OR Benin[Title/Abstract]) OR “Burkina Faso”[Title/Abstract]) OR “Cape Verde”[Title/Abstract]) OR “Cote d'Ivoire”[Title/Abstract]) OR “Ivory Coast”[Title/Abstract]) OR Gambia[Title/Abstract]) OR Ghana[Title/Abstract]) OR Guinea[Title/Abstract]) OR Guinea-Bissau[Title/Abstract]) OR Liberia[Title/Abstract]) OR Mali[Title/Abstract]) OR Mauritania[Title/Abstract]) OR Niger[Title/Abstract]) OR Nigeria[Title/Abstract]) OR Senegal[Title/Abstract]) OR “Sierra Leone”[Title/Abstract]) OR Togo[Title/Abstract]) OR Angola[Title/Abstract]) OR Cameroon[Title/Abstract]) OR “Central African Republic”[Title/Abstract]) OR Chad[Title/Abstract]) OR Congo[Title/Abstract]) OR “Equatorial Guinea”[Title/Abstract]) OR Gabon[Title/Abstract]) OR (“Sao Tome[Title/Abstract] AND Principe”[Title/Abstract])) OR Botswana[Title/Abstract]) OR Lesotho[Title/Abstract]) OR Namibia[Title/Abstract]) OR South Africa[Title/Abstract]) OR Swaziland[Title/Abstract]) |
| **Scopus** | TITLE-ABS-KEY("intestinal parasites" OR "intestinal parasite" OR "intestinal parasitic" "intestinal protozoa" OR "protozoan parasites" OR "gastrointestinal protozoa" OR "parasitic diarrhoea" OR "parasitic diarrhea" OR entamoeba OR giardia OR cryptosporidium OR balantidium OR cyclospora OR cystoisospora OR amebiasis OR amoebiasis OR giardiasis OR cryptosporidiosis OR balantidiasis OR cyclosporiasis OR cystoisosporiasis) AND TITLE-ABS-KEY(children OR paediatric OR pediatric) AND TITLE-ABS-KEY(Algeria OR Egypt OR Libya OR Morocco OR "South Sudan" OR Sudan OR Tunisia OR Burundi OR Comoros OR Djibouti OR Eritrea OR Ethiopia OR Kenya OR Madagascar OR Malawi OR Mauritius OR Mozambique OR Rwanda OR Seychelles OR Somalia OR Tanzania OR Uganda OR Zambia OR Zimbabwe OR Benin OR "Burkina Faso" OR "Cape Verde" OR "Cote d'Ivoire" OR "Ivory Coast" OR Gambia OR Ghana OR Guinea OR Guinea-Bissau OR Liberia OR Mali OR Mauritania OR Niger OR Nigeria OR Senegal OR "Sierra Leone" OR Togo OR Angola OR Cameroon OR "Central African Republic" OR Chad OR Congo OR "Equatorial Guinea" OR Gabon OR "Sao Tome and Principe" OR Botswana OR Lesotho OR Namibia OR "South Africa" OR Swaziland) |
| **Embase** | ('intestinal parasites':ab,ti OR 'intestinal parasite':ab,ti OR 'intestinal parasitic':ab,ti OR 'intestinal protozoa':ab,ti OR 'protozoan parasites':ab,ti OR 'gastrointestinal protozoa':ab,ti OR 'parasitic diarrhoea':ab,ti OR 'parasitic diarrhea':ab,ti OR entamoeba:ab,ti OR giardia:ab,ti OR cryptosporidium:ab,ti OR balantidium:ab,ti OR cyclospora:ab,ti OR cystoisospora:ab,ti OR amebiasis:ab,ti OR amoebiasis:ab,ti OR giardiasis:ab,ti OR cryptosporidiosis:ab,ti OR balantidiasis:ab,ti OR cyclosporiasis:ab,ti OR cystoisosporiasis:ab,ti) AND (children:ab,ti OR paediatric:ab,ti OR pediatric:ab,ti) AND (algeria:ab,ti OR egypt:ab,ti OR libya:ab,ti OR morocco:ab,ti OR 'south sudan':ab,ti OR sudan:ab,ti OR tunisia:ab,ti OR burundi:ab,ti OR comoros:ab,ti OR djibouti:ab,ti OR eritrea:ab,ti OR ethiopia:ab,ti OR kenya:ab,ti OR madagascar:ab,ti OR malawi:ab,ti OR mauritius:ab,ti OR mozambique:ab,ti OR rwanda:ab,ti OR seychelles:ab,ti OR somalia:ab,ti OR tanzania:ab,ti OR uganda:ab,ti OR zambia:ab,ti OR zimbabwe:ab,ti OR benin:ab,ti OR 'burkina faso':ab,ti OR 'cape verde':ab,ti OR 'cote divoire':ab,ti OR 'ivory coast':ab,ti OR gambia:ab,ti OR ghana:ab,ti OR guinea:ab,ti OR 'guinea bissau':ab,ti OR liberia:ab,ti OR mali:ab,ti OR mauritania:ab,ti OR niger:ab,ti OR nigeria:ab,ti OR senegal:ab,ti OR 'sierra leone':ab,ti OR togo:ab,ti OR angola:ab,ti OR cameroon:ab,ti OR 'central african republic':ab,ti OR chad:ab,ti OR congo:ab,ti OR 'equatorial guinea':ab,ti OR gabon:ab,ti OR 'sao tome principe':ab,ti OR botswana:ab,ti OR lesotho:ab,ti OR namibia:ab,ti OR 'south africa':ab,ti OR swaziland:ab,ti) |
| **Web of Science** | TI=("intestinal parasites" OR "intestinal parasite" OR "intestinal parasitic" OR "intestinal protozoa" OR "protozoan parasites" OR “gastrointestinal protozoa” OR “parasitic diarrhoea” OR “parasitic diarrhea” OR entamoeba OR giardia OR cryptosporidium OR balantidium OR cyclospora OR cystoisospora OR amebiasis OR amoebiasis OR giardiasis OR cryptosporidiosis OR balantidiasis OR cyclosporiasis OR cystoisosporiasis) AND TI=(children OR paediatric OR pediatric) AND TI=(Algeria OR Egypt OR Libya OR Morocco OR "South Sudan" OR Sudan OR Tunisia OR Burundi OR Comoros OR Djibouti OR Eritrea OR Ethiopia OR Kenya OR Madagascar OR Malawi OR Mauritius OR Mozambique OR Rwanda OR Seychelles OR Somalia OR Tanzania OR Uganda OR Zambia OR Zimbabwe OR Benin OR "Burkina Faso" OR "Cape Verde" OR "Cote d'Ivoire" OR "Ivory Coast" OR Gambia OR Ghana OR Guinea OR Guinea-Bissau OR Liberia OR Mali OR Mauritania OR Niger OR Nigeria OR Senegal OR "Sierra Leone" OR Togo OR Angola OR Cameroon OR "Central African Republic" OR Chad OR Congo OR "Equatorial Guinea" OR Gabon OR "Sao Tome and Principe" OR Botswana OR Lesotho OR Namibia OR "South Africa" OR Swaziland)  Indexes=SCI-EXPANDED, SSCI, A&HCI, CPCI-S, CPCI-SSH, BKCI-S, BKCI-SSH, ESCI Timespan=All years |
