# Supplementary material for: Prevalence of intestinal protozoan parasites among school children in africa: A systematic review and meta-analysis
Source: PLoS Negl Trop Dis. 2022 Feb 11;16(2):e0009971. doi: 10.1371/journal.pntd.0009971 (PMC8870593; doi:10.1371/journal.pntd.0009971)
Supplement: S2 Table — (DOCX) [file pntd.0009971.s003.docx]

**S2 Table.** Studies excluded after full text screening

| 1. Al-Shehri H, Stanton MC, LaCourse JE, et al. An extensive burden of giardiasis associated with intestinal schistosomiasis and anaemia in school children on the shoreline of Lake Albert, Uganda. Trans R Soc Trop Med Hyg. 2016;110:597-603. Same results published in 2019 2. Austin NI. Assessing geohelminth parasites among geophagous school children, in Owerri Metropolis Area, Imo State, South-Eastern Nigeria. International Journal of Infectious Diseases. 2016;45:352. Conference paper 3. Adewunmi CO, Gebremedhin G, Becker W, Olurunmola FO, Dorfler G, Adewunmi TA. Schistosomiasis and intestinal parasites in rural villages in southwest Nigeria: An indication for expanded programme on drug distribution and integrated control programme in Nigeria. Tropical Medicine and Parasitology. Published in 1993 4. Abah AE, Arene FO. Status of Intestinal Parasitic Infections among Primary School Children in Rivers State, Nigeria. J Parasitol Res. 2015;2015:937096. Only helminths have been reported. 5. Abidoye RO, Soroh KW. A study on the effects of urbanization on the nutritional status of primary school children in Lagos, Nigeria. Nutr Health. 1999;13:141-151. Published in 1999. 6. Adoubryn KD, Kouadio-Yapo CG, Ouhon J, Aka NA, Bintto F, Assoumou A. [Intestinal parasites in children in Biankouma, Ivory Coast (mountaineous western region): efficacy and safety of praziquantel and albendazole]. Med Sante Trop. 2012;22:170-176. Only helminths have been reported. 7. Adugna S, Kebede T, Mekonnen Z, Degarege A, Liang S, Erko B. Diagnostic performance of Mini Parasep(R) solvent-free faecal parasite concentrator relative to Kato-Katz and McMaster for the diagnosis of intestinal parasitic infections. Trans R Soc Trop Med Hyg. 2017;111:572-578. Evaluation of diagnostic test 8. Al-Herrawy AZ, Yamamah GA, Saleh FR, Gad MA. Detection and molecular identification of microsporidia in urine samples from school children in South Sinai, Egypt. Research Journal of Pharmaceutical, Biological and Chemical Sciences. 2016;7:1329-1337. The full text was not acceesed, and the corresponding author didnot respond to the email. 9. Anagha LI, Inegbenosun CU, Inegbenosun H. Prevalence of intestinal helminthic infections among secondary school students in Edo State, Nigeria. African Journal of Clinical and Experimental Microbiology. 2020;21:156-163. Only helminths have been reported. 10. Abd El-Latif, Naglaa Fathi, et al. "Molecular characterization of Giardia intestinalis detected in humans and water samples in Egypt." Acta parasitologica 65.2 (2020): 482-489. Pre-school Chlidren 11. Barda B, Ianniello D, Zepheryne H, et al. Parasitic infections on the shore of Lake Victoria (East Africa) detected by Mini-FLOTAC and standard techniques. Acta Tropica. 2014;137:140-146. The participants in the study are both adults and schoolchildren, making it impossible to collect data from solely youngsters 12. Barda BD, Rinaldi L, Ianniello D, et al. Mini-FLOTAC, an Innovative Direct Diagnostic Technique for Intestinal Parasitic Infections: Experience from the Field. PLoS Neglected Tropical Diseases. 2013;7. Development of diagnostic methods prevalence with 3 methods. 13. Bayoumy AMS, Ibrahim WLF, Abou El Nour BM, Said AAA. THE PARASITIC PROFILE AMONG SCHOOL CHILDREN IN EI-WADI EL-GADDED GOVERNORATE, EGYPT. J Egypt Soc Parasitol. 2016;46:605-612. Missing data on total sample size and overall prevalence, and the corresponding author didnot respond to the email. 14. Ben Musa N, Sehari A, Hawas A. Intestinal parasitic infections among school children in Tripoli, Libya. Journal of the Egyptian Society of Parasitology. 2007;37:1011-1016. The full text was not acceesed, and the corresponding author didnot respond to the email. 15. Ben Musa NA, Ibrahim R. Long term formalin preserved stool specimens for detection of intestinal parasites from school aged children in Tripoli, Libya. Journal of the Egyptian Society of Parasitology. 2007;37:1049-1054. Stool analysis done after 9 months from collection. 16. Burger PJ. The occurrence of intestinal parasites in school children in the Tygerberg area. South African Medical Journal. 1968;42:811-812. Published in 1968 17. Carvalho P, Teodósio R. Intestinal parasitic infection among schoolchildren in Fogo Island, Cape Verde. Tropical Medicine and International Health. 2011;16:195. The article was not found, similar title was accessed but from Thailand. 18. Chard AN, Trinies V, Moss DM, Chang HH, Freeman MC. Evaluating the impact of school water, sanitation and hygiene improvements using the presence of serum antibodies for enteric and neglected tropical diseases among school children in mali. American Journal of Tropical Medicine and Hygiene. 2016;95:564. The full text was not acceesed. 19. Coulibaly JT, Furst T, Silue KD, et al. Intestinal parasitic infections in schoolchildren in different settings of Cote d'Ivoire: effect of diagnostic approach and implications for control. Parasit Vectors. 2012;5:135. Difficult to extract the overall prevalence. 20. Coulibaly SO, Maïga M, Sawadogo M, Magnussen P, Guiguemde RT, Some I. Epidemiology of intestinal helminths, schistosomiasis andectoparasites in schoolchildren in Burkina Faso. Tropical Medicine and International Health. 2011;16:192. Only helminths have been reported. 21. Crotti D, Del Sante M, Fonzo G, Gebremariam T. Schistosomiasis and intestinal parasitosis among school-children in Mendefera, Eritrea. Giornale Italiano di Medicina Tropicale. 1999;4:15-19. . Published in 1999 22. Curtale F, Nabil M, el Wakeel A, Shamy MY. Anaemia and intestinal parasitic infections among school age children in Behera Governorate, Egypt. Behera Survey Team. J Trop Pediatr. 1998;44:323-328. Published in 1999. 23. Dacal E, Saugar JM, de Lucio A, et al. Prevalence and molecular characterization of Strongyloides stercoralis, Giardia duodenalis, Cryptosporidium spp., and Blastocystis spp. isolates in school children in Cubal, Western Angola. Parasit Vectors. 2018;11:67. Difficult to extract the overall prevalence. 24. Dana D, Vlaminck J, Mekonnen Z, et al. The clinical sensitivity of standard direct wet mount microscopy for soil-transmitted helminth infections in school children in Jimma, South-West Ethiopia. Transactions of the Royal Society of Tropical Medicine and Hygiene. 2019;113:S96. Only helminths have been reported. 25. Dancesco P, Abeu J, Akakpo C, et al. Intestinal parasitoses in a village of Côte d'Ivoire. I: Control and prevention plan. Cahiers Sante. 2005;15:5-10. 26. Desalegn A, Mossie A, Gedefaw L. Nutritional iron deficiency anemia: Magnitude and its predictors among school age children, southwest ethiopia: A community based cross-sectional study. PLoS ONE. 2014;9. Community based study 27. Dessie A, Gebrehiwot TG, Kiros B, Wami SD, Chercos DH. Intestinal parasitic infections and determinant factors among school-age children in Ethiopia: a cross-sectional study. BMC Res Notes. 2019;12:777. Difficult to extract the overall prevalence. 28. Develoux M, Mouchet F, Labo R. Intestinal parasitic diseases of school children in the Republic of Niger. Bulletin de la Societe de pathologie exotique et de ses filiales. 1986;79:571-575. The full text was not acceesed, and the corresponding author didnot respond to the email. 29. Di Cristanziano V, Santoro M, Parisi F, et al. Genetic characterization of Giardia duodenalis by sequence analysis in humans and animals in Pemba Island, Tanzania. Parasitol Int. 2014;63:438-441. The full text was not acceesed, and the corresponding author didnot respond to the email. 30. Dorkenoo MA, Yakpa K, Chard AN, et al. Prevalence of intestinal parasites in schoolaged children within the framework of the fight against neglected tropical diseases (NTDS) in the city of Lomé, Togo. American Journal of Tropical Medicine and Hygiene. 2014;91:345. The article was not found, similar title was accessed but from Argentina. 31. Efunshile MA, Ngwu BA, Kurtzhals JA, Sahar S, Konig B, Stensvold CR. Molecular Detection of the Carriage Rate of Four Intestinal Protozoa with Real-Time Polymerase Chain Reaction: Possible Overdiagnosis of Entamoeba histolytica in Nigeria. Am J Trop Med Hyg. 2015;93:257-262. Difficult to extract the overall prevalence. 32. El Fatni C, Olmo F, El Fatni H, Romero D, Rosales MJ. First genotyping of Giardia duodenalis and prevalence of enteroparasites in children from Tetouan (Morocco). Parasite. 2014;21. Difficult to extract the overall prevalence. 33. El Sahn FF, Deghedi BM, Mahdy NH, El Sahn A. The impact of intestinal parasitic infections on the nutritional status of primary school children in Alexandria, Egypt. The Journal of the Egyptian Public Health Association. 1997;72:113-151. The article was not found, similar title was accessed but from Argentina. 34. Enekwechi LC, Azubike CN. Survey of the prevalence of intestinal parasites in children of primary school age. West African journal of medicine. 1994;13:227-230. Published in 1994. 35. Erismann S, Diagbouga S, Schindler C, et al. School Children's Intestinal Parasite and Nutritional Status One Year after Complementary School Garden, Nutrition, Water, Sanitation, and Hygiene Interventions in Burkina Faso. Am J Trop Med Hyg. 2017;97:904-913. Intervention study, finding not clear. 36. Erismann S, Knoblauch AM, Diagbouga S, et al. High intestinal parasitic infections and malnutrition in school-aged children in Burkina Faso. Tropical Medicine and International Health. 2015;20:80. . Intervention study, finding not clear (duplicate). 37. Erismann S, Knoblauch AM, Diagbouga S, et al. Prevalence and risk factors of undernutrition among schoolchildren in the Plateau Central and Centre-Ouest regions of Burkina Faso. Infectious Diseases of Poverty. 2017;6., Same finding presented in study 34 (same author). 38. Erismann S, Shrestha A, Diagbouga S, et al. Complementary school garden, nutrition, water, sanitation and hygiene interventions to improve children's nutrition and health status in Burkina Faso and Nepal: a study protocol. BMC public health. 2016;16:244. interventions (a 1-year follow-up survey) 39. Erko B, Medhin G, Birrie H. Intestinal parasitic infections in Bahir Dar and risk factors for transmission. Tropical Medicine. 1995;37:73-78. Published in 1995. 40. Eziefule CM, Okaka CE. Gastro-intestinal parasitic infection amongst primary school children in Ogiobo and Eresoyen primary schools in Benin City, Edo state, Nigeria. Tropical Medicine and International Health. 2017;22:316. The article was not accessed. 41. Fentahun AA, Asrat A, Bitew A, Mulat S. Intestinal parasitic infections and associated factors among mentally disabled and non-disabled primary school students, Bahir Dar, Amhara regional state, Ethiopia, 2018: A comparative cross-sectional study. BMC Infectious Diseases. 2019;19. Comparing to group (mentally disabled and non-disabled students) 42. Forson AO, Arthur I, Ayeh-Kumi PF. The role of family size, employment and education of parents in the prevalence of intestinal parasitic infections in school children in Accra. PLoS ONE. 2018;13. same finding presented in study 19. Same finding presented in study 37 (same author). 43. Furnée CA, West CE, Van der Haar F, Hautvast JGAJ. Effect of intestinal parasite treatment on the efficacy of oral iodized oil for correcting iodine deficiency in schoolchildren. American Journal of Clinical Nutrition. 1997;66:1422-1427. Published in 1997. 44. Geus D, Sifft K, Fraundorfer K, et al. Extensive geographical variation in the prevalence of intestinal parasites in southern highland Rwanda. Tropical Medicine and International Health. 2015;20:316-317. The article was not found, but similar title was published by Hailegebriel, T, 2017. However, Geus D, 2019 was seen). 45. Geus D, Sifft KC, Habarugira F, et al. Co-infections with Plasmodium, Ascaris and Giardia among Rwandan schoolchildren. Trop Med Int Health. 2019;24:409-420. Difficult to extract the overall prevalence. 46. Guidetti C, Ricci L, Vecchia L. Aetiology of intestinal parasites in a sample of students from mozambique. Infezioni in Medicina. 2011;19:157-165. The article was not found, similar title was accessed but from Libya and Lebanon available. 47. Haller L, Lauber E. Health of schoolchildren in the Ivory Coast. Public health aspects: Nutrition and growth in connection with parasitic diseases. Acta Tropica. 1980; Published in 1980. 48. Heckendorn F, N'Goran EK, Felger I, et al. Species-specific field testing of Entamoeba spp. in an area of high endemicity. Trans R Soc Trop Med Hyg. 2002;96:521-528. Repeated samples. 49. Hojer B, Nordberg E. Health survey of a rural elementary school in Ethiopia. Ethiopian Medical Journal. 1973;11:75-92. Published in 1973. 50. Hori E. A survey of parasitic helminths and protozoa in Ife, Nigeria. Journal of Saitama Medical School. 1978;5:143-150. Published in 1978. 51. Houmsou RS, Amuta EU, Olusi TA. Prevalence of intestinal parasites among primary school children in Makurdi, Benue State-Nigeria. Internet Journal of Infectious Diseases. 2010;8. Difficult to extract the overall prevalence. 52. Hurlimann E, Houngbedji CA, N'Dri PB, et al. Effect of deworming on school-aged children's physical fitness, cognition and clinical parameters in a malaria-helminth co-endemic area of Cote d'Ivoire. BMC Infect Dis. 2014;14:411. 53. Kabatereine NB, Kemijumbi J, Kazibwe F, Onapa AW. Human intestinal parasites in primary school children in Kampala , Uganda. East African Medical Journal. 1997;74:311-314. 1997. Published in 1997. 54. Kassaw, M.W., Abebe, A.M., Tlaye, K.G. et al. Prevalence and risk factors of intestinal parasitic infestations among preschool children in Sekota town, Waghimra zone, Ethiopia. BMC Pediatr 19, 437 (2019). Preschool children 55. Kamga HLF, Nde PF, Fomumbod SA, et al. The relationship between perception and prevalence of faecal-orally transmitted parasitic infections among school children in a rural community in Cameroon. African Journal of Clinical and Experimental Microbiology. 2013;14:184-189. Multi phases 56. Kebede A, Verweij JJ, Endeshaw T, et al. The use of real-time PCR to identify Entamoeba histolytica and E. dispar infections in prisoners and primary-school children in Ethiopia. Ann Trop Med Parasitol. 2004;98:43-48. The participants in the study are both prisoners and schoolchildren, making it impossible to collect data from solely school children. 57. Ketema H, Biruksew A, Mekonnen Z. Prevalence of Necator americanus infection and risk factors among school-age children in Mirab Abaya District, South Ethiopia. Asian Pacific Journal of Tropical Disease. 2015;5:363-368. Only helminths have been reported. 58. Labib JR, El Shafei AM, Haggag AA. The clinical profile of students attending informal schools in three rural governorates in upper Egypt: a community-based study. The Journal of the Egyptian Public Health Association. 2013;88:153-159. Only helminths have been reported. 59. Loewenson R, Mason PR, Patterson BA. Giardiasis and the nutritional status of Zimbabwean schoolchildren. Ann Trop Paediatr. 1986;6:73-78. Published in 1986. 60. Magambo JK, Zeyhle E, Wachira TM. Prevalence of intestinal parasites among children in southern Sudan. East African Medical Journal. 1998;75:288-290. Published in 1998. 61. Mohamed N, Muse A, Wordofa M, et al. Increased prevalence of cestode infection associated with history of deworming among primary school children in Ethiopia. American Journal of Tropical Medicine and Hygiene. 2019;101:641-649. Difficult to extract the overall prevalence. 62. Mohammed K, Abdullah MR, Omar J, Ifeanyi IE, Ismail A. Intestinal parasitic infection and assessment of risk factors in North-Western, Nigeria: A community based study. International Journal of Pharma Medicine and Biological Sciences. 2015;4:141-145. Community based study. 63. Mukhtar AAZ, Abbas MI, Sanyal RK. Intestinal parasitic load in school-going children of East Equitoria province of Sudan. Journal of Communicable Diseases. 1981;13:200-203. Published in 1981. 64. Nour BYM, El Emam NH, Abakar AD. Laboratory diagnosis and risk factors of gastrointestinal parasites among basic school children in greater Wad Madani locality, Gezira State, Sudan. Tropical Medicine and International Health. 2015;20:439. The full text was not acceesed, and the corresponding author didnot respond to the email. 65. Nute AW, Endeshaw T, Stewart AEP, et al. Prevalence of soil-transmitted helminths and Schistosoma mansoni among a population-based sample of school-age children in Amhara region, Ethiopia. Parasit Vectors. 2018;11:431. Only STHs were reported. 66. Nxasana N, Baba K, Bhat V, Vasaikar S. Prevalence of intestinal parasites in primary school children of mthatha, eastern cape province, South Africa. Ann Med Health Sci Res. 2013;3:511-516. Difficult to extract the overall prevalence. 67. Ojurongbe O, Adegbayi AM, Bolaji OS, Akindele AA, Adefioye OA, Adeyeba OA. Asymptomatic falciparum malaria and intestinal helminths co-infection among school children in Osogbo, Nigeria. Journal of Research in Medical Sciences. 2011;16. Only STHs were reported. 68. Okafor JI, Okunji PO. Prevalence of Cryptosporidium oocysts in faecal samples of some school children in Enugu State, Nigeria. J Commun Dis. 1996;28:49-55. Published in 1996. 69. Okpala I. The incidence of intestinal parasites among school children in Lagos (Nigeria). West Afr Med J. 1956;5:167-170. Published in 1956. 70. Olopade BO, Idowu CO, Oyelese AO, Aboderin AO. INTESTINAL PARASITES, NUTRITIONAL STATUS AND COGNITIVE FUNCTION AMONG PRIMARY SCHOOL PUPILS IN ILE-IFE, OSUN STATE, NIGERIA. Afr J Infect Dis. 2018;12:21-28. Only STHs were reported. 71. Osisiogu FUO, Nwoke BEB, Ukaga CN, Amaechi AA, Ezeigbo OR, Amadi CAI. Prevalence of intestinal parasites and bacteria among school pupils in aba, abia state. Nigerian Journal of Parasitology. 2018;39:74-78. The full text was not acceesed, and the corresponding author didnot respond to the email. 72. Ostwald R, Fitch M, Arnhold R. The effect of intestinal parasites on nutritional status in well-nourished school-age children in the highlands of Papua New Guinea. Nutrition Reports International. 1984;30:1409-1421. Only STHs were reported. 73. Premaletha T, Thomas K, Babu PK. Helminthiasis and anemia among school children. Indian Journal of Public Health Research and Development. 2016;7:95-99. The full text was not acceesed, and the corresponding author didnot respond to the email. 74. Roma, Belay, and Solomon Worku. "Magnitude of Schistosoma mansoni and intestinal helminthic infections among school children in Wondo-Genet zuria, southern Ethiopia." The Ethiopian Journal of Health Development 11.2 (1997). Published in 1997. 75. Raso G, Utzinger J, Silue KD, et al. Disparities in parasitic infections, perceived ill health and access to health care among poorer and less poor schoolchildren of rural Cote d'Ivoire. Trop Med Int Health. 2005;10:42-57. Difficult to extract the overall prevalence. 76. Rebollo M, Tchuenté T, Perea C, Polentinos E, Bueno A, Sobrón B. Prevalence of soil transmitted helminthiasis on school age children in south Cameroon. Tropical Medicine and International Health. 2011;16:223. Only helminths have been reported. 77. Rekart ML, Plastino J, Carr C. Health status of teenage school boys in eastern Sudan. East Afr Med J. 1985;62:54-59. Published in 1985. 78. Sadaga GA, Kassem HH. Prevalence of intestinal parasites among primary schoolchildren in Derna District, Libya. Journal of the Egyptian Society of Parasitology. 2007;37:205-214. Only abstract accessible, the full article requested via researchgate but no response. 79. Samie A, Bessong PO, Obi CL, et al. Cryptosporidium species: preliminary descriptions of the prevalence and genotype distribution among school children and hospital patients in the Venda region, Limpopo Province, South Africa. Exp Parasitol. 2006;114:314-322. The full text was not acceesed, and the corresponding author didnot respond to the email. 80. Sargeaunt PG, Williams JE, Jackson TF, Simjee AE. A zymodeme study of Entamoeba histolytica in a group of South African schoolchildren. Trans R Soc Trop Med Hyg. 1982;76:401-402. Published in 1982. 81. Schwarz NG, Hagen RM, Heriniaina JN, et al. Stool examination of 410 asymptomatic school children in a highland village of Madagascar. Tropical Medicine and International Health. 2013;18:62. Conference paper. 82. Selim SG, Attia AA, Mahmoud SA, Ismail AI. Behavioral risk factors associated with intestinal parasitic infestations among children in rural areas. Indian Journal of Public Health Research and Development. 2019;10:2140-2145. Paired sample, mother and their children. 83. Shimada M, Nojima H, Hirata M, et al. Intestinal parasitic infections of school children in Kwale district of coast province, Kenya. Tropical Medicine. 1997;39:57-64. Published in 1997. 84. Siddig HS, Mohammed IA, Mohammed MN, Bashir AM. Prevalence of Intestinal Parasites among Selected Group of Primary School Children in Alhag Yousif Area, Khartoum, Sudan. International Journal of Medical Research & Health Sciences. 2017;6:125-131. Difficult to extract the overall prevalence. 85. Singer MN, Malhotra I, Mutuku F, et al. Hookworm infection in school-aged Kenyan children is associated with lower physical fitness. American Journal of Tropical Medicine and Hygiene. 2014;91:523. Only helminths have been reported. 86. Sitotaw B, Mekuriaw H, Damtie D. Prevalence of intestinal parasitic infections and associated risk factors among Jawi primary school children, Jawi town, north-west Ethiopia. BMC Infectious Diseases. 2019;19. Difficult to extract the overall prevalence. 87. Speich B, Marti H, Ame SM, et al. Prevalence of intestinal protozoa infection among school-aged children on Pemba Island, Tanzania, and effect of single-dose albendazole, nitazoxanide and albendazole-nitazoxanide. Parasit Vectors. 2013;6, Intervention. 88. Steenhard NR, Ornbjerg N, Molbak K. Concurrent infections and socioeconomic determinants of geohelminth infection: a community study of schoolchildren in periurban Guinea-Bissau. Trans R Soc Trop Med Hyg. 2009;103:839-845. Community based study. 89. Tariku EZ, Abebe GA, Melketsedik ZA, et al. Anemia and its associated factors among school-age children living in different climatic zones of Arba Minch Zuria District, Southern Ethiopia. BMC Hematol. 2019;19:6. Only STHs were reported. 90. Tefera E, Belay T, Mekonnen SK, Zeynudin A, Belachew T. Prevalence and intensity of soil transmitted helminths among school children of Mendera Elementary School, Jimma, Southwest Ethiopia. Pan Afr Med J. 2017;27:88. Only helminths have been reported. 91. Teklemariam D, Legesse M, Degarege A, Liang S, Erko B. Schistosoma mansoni and other intestinal parasitic infections in schoolchildren and vervet monkeys in Lake Ziway area, Ethiopia. BMC Res Notes. 2018;11:146. Only helminths have been reported. 92. Tembo SJ, Mutengo MM, Sitali L, et al. Prevalence and genotypic characterization of Giardia duodenalis isolates from asymptomatic school-going children in Lusaka, Zambia. Food Waterborne Parasitol. 2020;19:e00072. Only abtract is accessible. 93. Tomlinson M, Adams V, Chopra M, Jooste P, Strydom E, Dhansay A. Survey of iodine deficiency and intestinal parasitic infections in school-going children: Bie Province, Angola. Public Health Nutrition. 2010;13:1314-1318. Only abtract is accessible. 94. Tongjura JDC, Ombugadu JR, Abdullahi MM, Blessing MA, Amuga GA, Mafuyai HB. Intestinal parasites amongst primary school children attending Ta’al model primary school in Lafia Local Government Area of Nasarawa State, Nigeria. Nigerian Journal of Parasitology. 2019;40:92-96. The full text was not acceesed, and the corresponding author didnot respond to the email. 95. Tosson, Morsy A, Farrag AM, Sabry AH, Salama MM, Arafa MA. Ecto and endoparasites in two primary schools in Qualyob City, Egypt. J Egypt Soc Parasitol. 1991;21:391-401. Published in 1991. 96. Traore SG, Odermatt P, Bonfoh B, et al. No Paragonimus in high-risk groups in Cote d'Ivoire, but considerable prevalence of helminths and intestinal protozoon infections. Parasit Vectors. 2011;4:96. Participants are Patients. 97. Tulu B, Taye S, Amsalu E. Prevalence and its associated risk factors of intestinal parasitic infections among Yadot primary school children of South Eastern Ethiopia: A cross-sectional study. BMC Research Notes. 2014;7. Difficult to extract the overall prevalence. 98. Tulu B, Taye S, Zenebe Y, Amsalu E. Intestinal Parasitic Infections and Nutritional Status among Primary School Children in Delo-mena District, South Eastern Ethiopia. Iran J Parasitol. 2016;11:549-558. Difficult to extract the overall prevalence. 99. Ugbomoiko US, Dalumo V, Ofoezie IE, Obiezue RN. Socio-environmental factors and ascariasis infection among school-aged children in Ilobu, Osun State, Nigeria. Trans R Soc Trop Med Hyg. 2009;103:223-228. Only STHs were reported. 100. Urbani C, Touré A, Hamed AO, et al. Intestinal parasitic infections and schistosomiasis in the valley of the Senegal river in the Islamic Republic of Mauritania. Médecine tropicale : revue du Corps de santé colonial. 1997;57:157-160. Published in 1997. 101. Utzinger J, Muller I, Vounatsou P, Singer BH, N'Goran EK, Tanner M. Random spatial distribution of Schistosoma mansoni and hookworm infections among school children within a single village. J Parasitol. 2003;89:686-692. (Community based study. 102. Utzinger J, N'Goran EK, Esse Aya CM, et al. Schistosoma mansoni, intestinal parasites and perceived morbidity indicators in schoolchildren in a rural endemic area of western Cote d'ivoire. Tropical Medicine and International Health. 1998;3:711-720 Published in 1998. 103. Weldesenbet H, Worku A, Shumbej T. Prevalence, infection intensity and associated factors of soil transmitted helminths among primary school children in Gurage zone, South Central Ethiopia: a cross-sectional study design. BMC Res Notes. 2019;12:231. Only helminths have been reported. 104. Worku N, Erko B, Torben W, et al. Malnutrition and intestinal parasitic infections in school children of Gondar, North West Ethiopia. Ethiopian Medical Journal. 2009;47:9-16. The full text was not acceesed, and the corresponding author didnot respond to the email. 105. Yami A, Mamo Y, Kebede S. Prevalence and predictors of intestinal helminthiasis among school children in jimma zone; a cross-sectional study. Ethiop J Health Sci. 2011;21:167-174. Only helminths have been reported. 106. Yap P, Muller I, Walter C, et al. Disease, activity and schoolchildren's health (DASH) in Port Elizabeth, South Africa: a study protocol. BMC Public Health. 2015;15:1285 2. A 2-year cohort study 107. Yimer M, Hailu T, Mulu W, Abera B. Evaluation performance of diagnostic methods of intestinal parasitosis in school age children in Ethiopia. BMC Res Notes. 2015;8:820. evaluation of diagnostic kits. |
| --- |
