# Supplementary material for: Prevalence of intestinal protozoan parasites among school children in africa: A systematic review and meta-analysis
Source: PLoS Negl Trop Dis. 2022 Feb 11;16(2):e0009971. doi: 10.1371/journal.pntd.0009971 (PMC8870593; doi:10.1371/journal.pntd.0009971)
Supplement: S3 Table — (DOCX) [file pntd.0009971.s004.docx]

| **S3 Table**. Quality assessment of the included studies | | | | | | | | | | | |
| --- | --- | --- | --- | --- | --- | --- | --- | --- | --- | --- | --- |
| **No.** | **Study ID** | **Questions assessing included studies** | | | | | | | | | **Yes (%)** |
|  |  | **1** | **2** | **3** | **4** | **5** | **6** | **7** | **8** | **9** |  |
| 1 | Abdel-Aziz 2010 | Y | N | N | N | Y | Y | Y | Y | N | 55.6 |
| 2 | Abossie 2014 | Y | Y | Y | Y | Y | Y | Y | Y | Y | 100.0 |
| 3 | Adams 2005 | Y | Y | U | Y | Y | Y | Y | Y | U | 77.8 |
| 4 | Adedoja 2015 | Y | Y | U | Y | Y | Y | Y | Y | U | 77.8 |
| 5 | Alemu 2019a | Y | Y | Y | Y | Y | Y | Y | Y | Y | 100.0 |
| 6 | Alemu, 2019b | Y | Y | Y | N | Y | Y | Y | Y | N | 77.8 |
| 7 | Al-Shehri 2019 | Y | U | N | N | Y | Y | Y | Y | N | 55.6 |
| 8 | Amare 2013 | Y | Y | Y | Y | Y | U | U | N | Y | 66.7 |
| 9 | Awolaju 2009 | Y | Y | N | Y | Y | Y | Y | Y | N | 77.7 |
| 10 | Ayogu 2015 | Y | N | Y | Y | N | Y | Y | N | Y | 66.7 |
| 11 | Baba 2012 | Y | N | Y | Y | Y | Y | Y | N | U | 66.7 |
| 12 | Birhanu 2018 | Y | Y | Y | Y | N | Y | Y | Y | Y | 88.9 |
| 13 | Bisangamo 2017 | Y | U | U | Y | Y | Y | Y | Y | U | 66.7 |
| 14 | Chege 2020 | Y | Y | U | Y | Y | N | N | Y | N | 55.6 |
| 15 | de Alegria 2017 | Y | U | Y | Y | Y | Y | Y | Y | N | 77.8 |
| 16 | Dyab 2016 | Y | Y | N | N | Y | Y | Y | Y | U | 66.7 |
| 17 | Erismann 2016 | Y | U | N | Y | Y | Y | Y | Y | U | 66.7 |
| 18 | Eyamo 2019 | Y | Y | N | Y | Y | Y | Y | Y | U | 77.7 |
| 19 | Fan 2012 | Y | N | N | N | Y | Y | Y | Y | U | 55.6 |
| 20 | Forson 2017 | Y | N | Y | Y | Y | Y | Y | Y | N | 77.8 |
| 21 | Gebretsadik 2020 | Y | Y | Y | Y | Y | Y | Y | Y | Y | 100.0 |
| 22 | Gelaw 2013 | Y | Y | Y | Y | Y | Y | Y | Y | N | 88.9 |
| 23 | Gyang 2019 | Y | Y | Y | Y | Y | Y | Y | N | Y | 88.9 |
| 24 | Hailegebriel 2018 | Y | Y | Y | Y | Y | Y | Y | Y | N | 88.9 |
| 25 | Hailegebriel 2017 | Y | Y | Y | Y | Y | Y | Y | Y | N | 88.9 |
| 26 | Hall 2008 | Y | Y | Y | Y | Y | Y | Y | Y | Y | 100.0 |
| 27 | Heimer 2015 | Y | N | N | Y | Y | Y | Y | Y | U | 66.7 |
| 28 | Htun 2018 | Y | N | N | Y | Y | N | Y | Y | U | 55.6 |
| 29 | Ibrahium 2011 | Y | N | N | Y | Y | Y | Y | Y | N | 66.7 |
| 30 | Ihejirika 2019 | Y | Y | Y | Y | Y | Y | Y | Y | Y | 100.0 |
| 31 | Jejaw 2015 | Y | Y | Y | Y | Y | Y | Y | N | U | 77.8 |
| 32 | Kesete 2020 | Y | Y | Y | Y | Y | Y | Y | Y | Y | 100 |
| 33 | Khaled 2020 | Y | N | U | Y | Y | Y | Y | Y | U | 66.7 |
| 34 | Legesse 2010 | Y | Y | Y | Y | Y | Y | Y | Y | Y | 100.0 |
| 35 | Liao 2016 | Y | N | N | Y | Y | Y | Y | N | N | 55.6 |
| 36 | Mahmud 2013 | Y | Y | U | Y | Y | Y | Y | N | U | 66.7 |
| 37 | Müller 2016 | Y | U | N | Y | Y | Y | Y | Y | U | 66.7 |
| 38 | Nguyen 2012 | Y | Y | Y | Y | Y | Y | Y | Y | Y | 100.0 |
| 39 | Njambi 2020 | Y | Y | U | Y | Y | Y | Y | Y | U | 77.8 |
| 40 | Oliveira 2015 | Y | N | Y | Y | N | Y | Y | Y | Y | 77.8 |
| 41 | Opara 2012 | Y | Y | N | Y | Y | Y | Y | Y | U | 77.8 |
| 42 | Orish 2019 | Y | N | Y | Y | N | Y | Y | Y | Y | 77.8 |
| 43 | Reji 2011 | Y | Y | Y | Y | Y | Y | Y | Y | Y | 100.0 |
| 44 | Sitotaw 2020 | Y | Y | Y | Y | Y | Y | Y | Y | Y | 100.0 |
| 45 | Tagajdid 2012 | Y | N | U | Y | Y | Y | Y | Y | N | 66.7 |
| 46 | Tembo 2020 | Y | N | N | Y | Y | Y | Y | Y | N | 66.7 |
| 1. Was the sample frame appropriate to address the target population? 2. Were study participants sampled in an appropriate way? 3. Was the sample size adequate? 4. Were the study subjects and the setting described in detail? 5. Was the data analysis conducted with sufficient coverage of the identified sample? 6. Were valid methods used for the identification of the condition? 7. Was the condition measured in a standard, reliable way for all participants? 8. Was there appropriate statistical analysis? 9. Was the response rate adequate, and if not, was the low response rate managed appropriately? Y=Yes; N=No; U=Unclear or NA Not applicable | | | | | | | | | | | |
