# Supplementary material for: Prevalence of intestinal protozoan parasites among school children in africa: A systematic review and meta-analysis
Source: PLoS Negl Trop Dis. 2022 Feb 11;16(2):e0009971. doi: 10.1371/journal.pntd.0009971 (PMC8870593; doi:10.1371/journal.pntd.0009971)
Supplement: S1 Fig — Subgroup analyses. Prevalence of intestinal protozoan infections among school children in Africa based on children enrolment time (A-C), different regions (D-G), countries (H-Z), diagnostic methods (AA-AC) and species (AD-AG). (DOCX) [file pntd.0009971.s005.docx]

**A**

**B**

**C**

**D**

**E**

**F**

**G**

**H**

**I**

**J**

**K**

**L**

**M**

**N**

**O**

**P**

**Q**

**R**

**S**

**T**

**U**

**V**

**W**

**X**

**Y**

**Z**

**AA**

**AB**

**AC**

**AD**

**AE**

**AF**

**AG**

**S1 Fig**. Subgroup analyses. Prevalence of intestinal protozoan infections among school children in Africa based on children enrolment time (A-C), different regions (D-G), countries (H-Z), diagnostic methods (AA-AC) and species (AD-AG).
