# Supplementary material for: Prevalence of intestinal protozoan parasites among school children in africa: A systematic review and meta-analysis
Source: PLoS Negl Trop Dis. 2022 Feb 11;16(2):e0009971. doi: 10.1371/journal.pntd.0009971 (PMC8870593; doi:10.1371/journal.pntd.0009971)
Supplement: S2 Fig — Sensitivity analysis by (A) excluding small studies, (B) excluding low- and moderate-quality studies, (C) excluding studies used non-microscopic diagnostic methods and (D) excluding outlier studies. (DOCX) [file pntd.0009971.s006.docx]

**A**

**B**

**C**

**D**

**S2 Fig.** Sensitivity analysis by A) excluding small studies, B) excluding low- and moderate-quality studies, C) excluding studies used non-microscopic diagnostic methods and D) excluding outlier studies.
