# Supplementary material for: Prevalence of intestinal protozoan parasites among school children in africa: A systematic review and meta-analysis
Source: PLoS Negl Trop Dis. 2022 Feb 11;16(2):e0009971. doi: 10.1371/journal.pntd.0009971 (PMC8870593; doi:10.1371/journal.pntd.0009971)
Supplement: Attachment — Submitted filename: Review report 07072021.pdf [file pntd.0009971.s007.pdf]

Review report 07/07/ 2021

Title: Prevalence of intestinal protozoan parasites among 29,968 school children in Africa: A systematic review and meta-analysis

General comments

Information provided in this manuscript is important, given the fact that there are no systematic review articles for the African continent on prevalence of intestinal protozoan parasites among school children. Results from this manuscript will clarify the current situation in the continent especially for implementing prevention and control measures. However, there are several limitations which will need to be addressed as follows:

1. The data in this manuscript is from 19 African countries only, out of 54 African countries. Furthermore, over 50% of the data is from only three countries namely Ethiopia, Nigeria, and South Africa. How can authors generalise this data for the whole continent? Authors may need to change the title and reflect what is presented.
2. On line 32 authors stated that "Relevant studies published between January 2000 and December 2020" and on line 215, "A total of 29,968 school children aged 6–17 years were examined for the presence of IPPs from 2000 to 2020". But the data in the manuscript is from the year 2005, because papers from year 2000-2004 were excluded. It should be stated throughout the manuscript that the data is from the year 2005 and not from year 2000.
3. On line 252-253: "The prevalence of IPPs was remarkably increased from 0.2% during the period between 2005 and 2008 to 26.4% in the next four years (2009–2012). Again, from the year 2005 to 2008 only two studies from two countries were analysed i.e., South Africa and Ethiopia. These are the two countries which consistently reported low prevalence of IPPs, therefore stating that there is an increase of prevalence in the whole African region may not be the case because data is from countries with low prevalence.
4. It is well known that microscopy cannot differentiate between *E. histolytica* and non-pathogenic Entamoeba, hence reporting *E. histolytica* using microscopy may overestimate the magnitude of *E. histolytica*. This being the case, what is reported in this manuscript could also be an overestimation since most of the studies used microscopy as a diagnostic tool. This need to be mentioned in the limitation

Specific minor comments

1.Title: Delete the sample size in the title

2. Discussion on line 297-298: "The current review compiled eligible data on the prevalence of IPPs from 29,968 school children reported in **19 articles"** Change 19 articles to 19 countries.
3. Discussion line 308-309: Why do you start comparing with Iran which is one country, instead of comparing your data with data from another continental data.
