# Supplementary material for: Prevalence of intestinal protozoan parasites among school children in africa: A systematic review and meta-analysis
Source: PLoS Negl Trop Dis. 2022 Feb 11;16(2):e0009971. doi: 10.1371/journal.pntd.0009971 (PMC8870593; doi:10.1371/journal.pntd.0009971)
Supplement: Attachment — Submitted filename: 0. Response to the reviewers.pdf [file pntd.0009971.s008.pdf]

Response to Reviewer 1

Methods

Point 1: The objectives of the study are clearly articulated, however is not clear why the authors choose to exclude the helminths from the analysis. The authors indicated a research question, however a clear testable hypothesis is lacking. Which undermine the scientific impact of the results. Why is important to identify the pooled prevalence of intestinal protozoans in Africa? Please indicate the inclusion criteria for the meta-analysis; e.g. why small studies (N < 200) were included in the pooled prevalence?

Response 1: Many thanks for your comment.

- The authors would like to clarify that the primary objective of this systematic review and meta-analysis (SRMA) was to investigate all the intestinal parasites (Protozoa + helminths), but we found that including the names of all intestinal parasites will affect the accuracy of the online database search due to the limitation of the electronic databases, so helminths will be reported in another SRMA that will be conducted by our group as well.
- The study's main hypothesis was that the prevalence of intestinal protozoan parasites (IPPs) among school children in Africa is still high, despite the significant improvements in intestinal parasitic diseases identification and mass treatment, as well as in quality of life.
- Given that most clinicians and public health professionals may not have the time to find all of the original studies, critically evaluate them, and gather the evidence they want for their queries, systematic reviews may be their greatest source of evidence. As a result, pooling prevalence data on intestinal protozoans in Africa will be useful for understanding the IPPs status among African children and making relevant information more accessible to decision and policymakers. The findings of this study provide additional evidence that the burden of these parasites is still alarming in Africa.
- The inclusion and exclusion criteria have been extensively described under the section "Inclusion and exclusion criteria". We hope it's alright now (Line 139-147).
- Although sample size was not an inclusion or exclusion criteria, four small studies were excluded during the sensitivity analysis only. The results exhibited that omitting small studies (n < 200) had no significant effect on the summary of the pooled estimates, and the prevalence rate remained within the respective overall prevalence's 95% CI (from 25.8% to 23.9%).

Point 2: Is the study design appropriate to address the stated objectives? Yes, however only cross-sectional studies were included and surveys in hospitals were excluded. I would suggest not to exclude studies according to study design and to use the design as a stratification criteria in analyses. It is worth to check how many studies were excluded by the authors due to design criteria. Moreover, the authors classified the results and meta-analyses by year of publication. Instead, it is more relevant to extract and analyze the studies according to the period of data to show a potential evolution of the prevalence of IPPs especially of some species.

Response 2: Many thanks for your comment.

- The objective of this SRMA was to determine the prevalence of IPPs among school children that is why study conducted in hospital or community level were excluded.
- As the cross-sectional studies are the most appropriate study designs for estimating prevalence, including other design would not directly answer our research question rather than incorporating high levels of selection bias. We hope it's understandable now.
- Based on the reviewer's excellent suggestion, in the revised manuscript, we have classified and analysed the data based on the time period of the studies (table 2 and supplementary figure 1). We hope it's alright now.

Results

Few recommendations to consider.

Point 3: In Fig 2 review the classes descriptions, there are overlapping for the values 45, 60 and 75.

Response 3: Many thanks for identifying a crucial issue. We sincerely apologise for the typo; the figure and description have been revised**.** We hope it's alright now.

Point 4: For fig 3, would be interesting to add the study location after the study ID.

Response 4: Many thanks for your suggestion. We have added study locations followed by the author IDs. We hope the figure is alright now.

Point 5: In Table 1 DWN stands for Direct Wet Mount? Please add it to the footnote and correct in line 10.

Response 5: We apologies for missing this abbreviation. We have included the abbreviation at the footnote of Table 1.

Point 6: Add in table 3 the 95% CI for the difference of pooled prevalence compared to the main results.

Response 6: Many thanks for your suggestion. We have added the 95% differences of pooled prevalence compared to the main results at the Table 3.

Point 7: S1 Figure AH is it Escherichia coli or Entanmoeba coli?; AI is it Cryptosporidium parvum or Cryptosporidium spp?

Response 7: We apologies for the unintentional error and many thanks for pointing out the mistake. The supplementary figure has been updated as *Entamoeba coli* and *Cryptosporidium* spp. Thank you.

Point 8: Line 352, is described that nine types of protozoan were identified, kindly indicate each one of them in the results.

Response 8: The nine types were indicated in the result section (characteristics of included studies) line 2017-219. Thank you.

Response to Reviewer 2

General comments

Information provided in this manuscript is important, given the fact that there are no systematic review articles for the African continent on prevalence of intestinal protozoan parasites among school children. Results from this manuscript will clarify the current situation in the continent especially for implementing prevention and control measures. However, there are several limitations which will need to be addressed as follows:

Response: Many thanks for your kind words and the critical review

Point 1: The data in this manuscript is from 19 African countries only, out of 54 African countries. Furthermore, over 50% of the data is from only three countries namely Ethiopia, Nigeria, and South Africa. How can authors generalise this data for the whole continent? Authors may need to change the title and reflect what is presented.

Response 1: The authors strongly agree with this point, the major limitation of this study is the large gap in studies conducted in most African countries. The title, on the other hand, reflects the protocol and the methodology of this systematic review and meta-analysis (SRMA). All the 54 African countries were included in the search strategy, however articles from only 19 countries were identified to meet the inclusion criteria. Indeed, the findings of this SRMA may not accurately reflect the true prevalence status of the intestinal protozoan parasite among children in Africa, but they highlight the large gap in studies conducted in most African countries, highlighting the need for additional studies with different inclusion/exclusion criteria, as well as scholars focusing on intestinal protozoan parasites (IPPs) among school children in these countries. This point has previously been addressed in the discussion section as well as in the study limitation. We hope it's clear now. Many thanks for your comment.

Point 2: On line 32 authors stated that "Relevant studies published between January 2000 and December 2020" and on line 215, "A total of 29,968 school children aged 6–17 years were examined for the presence of IPPs from 2000 to 2020". But the data in the manuscript is from the year 2005, because papers from year 2000-2004 were excluded. It should be stated throughout the manuscript that the data is from the year 2005 and not from year 2000.

Response 2: The authors would like to clarify that the systematic online search on the four databases was limited to studies published between January 2000 and December 2020, however no study published between 2000 and 2004 met the inclusion criteria. Therefore, the statement in line 32 describes the methods of the online search, while the statement in line 215 describes the characteristics of the included studies. To minimise any misunderstanding for the reader, we deleted the time period in line 215. I hope this makes sense now. Thank you.

Point 3: On line 252-253: "The prevalence of IPPs was remarkably increased from 0.2% during the period between 2005 and 2008 to 26.4% in the next four years (2009–2012). Again, from the year 2005 to 2008 only two studies from two countries were analysed i.e., South Africa and Ethiopia. These are the two countries which consistently reported low prevalence of IPPs, therefore stating that there is an increase of prevalence in the whole African region may not be the case because data is from countries with low prevalence.

Response 3: We agree with the reviewer; however, in response to another reviewer's suggestion, the prevalence was reanalysed based on the period of study rather than the publication year; hence, this statement is no longer included in the manuscript; nevertheless, we would like to thank you for the insightful comment.

Point 4: It is well known that microscopy cannot differentiate between *E. histolytica* and non-pathogenic Entamoeba, hence reporting *E. histolytica* using microscopy may overestimate the magnitude of *E. histolytica*. This being the case, what is reported in this manuscript could also be an overestimation since most of the studies used microscopy as a diagnostic tool. This need to be mentioned in the limitation
Response 4: Many thanks for your comment. A similar statement has been added to the limitation part of the manuscript (Line 371-375). We hope it's alright now.

Specific minor comments

Point 5: Title: Delete the sample size in the title
Response 5: The sample size mentioned in the title has been removed. Thank you.

Point 2: Discussion on line 297-298: "The current review compiled eligible data on the prevalence of IPPs from 29,968 school children reported in **19 articles"** Change 19 articles to 19 countries.
Response 2: The sentence was revised accordingly (Line 297). Thank you.

Point 3: Discussion line 308-309: Why do you start comparing with Iran which is one country, instead of comparing your data with data from another continental data.
Response 3: The authors strongly agree with this point. The authors prioritised identifying similar research (systematic reviews and meta-analyses) that synthesised data on prevalence of IPPs among school children within other continents. However, based on our advanced search results, such references were very limited which is why our data was compared with country based systematic reviews. Thank you.

Response to Reviewer 3

Point 1: please add more data to forest plots such as period of data collection or by prevalence. Sorting alphabetically makes the results hard to be interpreted visually.
Response 1: The forest plots was revised accordingly. We have added new information on the weight of the studies and also countries the studies conducted in. In addition, we have sorted the studies based on the prevalence which was also the suggestion of another reviewer. All the additional forest plots in the supplementary files have also been updated as well. We hope it's alright now. Thank you.

Point 2: Fig 1: please recalculate the totals according to exclusions, they don't match. Please indicate reasons of exclusion of full-texts reviewed and make the list available.
Response 2: We sincerely apologise for the mistake. Figure 1 was updated in the revised manuscript. In addition, we have made a list of excluded studies with reasons as a supplementary file. We hope it's alright now. Thank you.

Point 3: Table 1: Please add %, report gender among cases (if available), it is probably more interesting than in the population. Add age of the population, treatment, delay to diagnostic, symptoms..etc.
Response 3: Many thanks for your suggestions. In the revised manuscript (Table 1), we have added the % of female apart from the sample size. Thank you.

Point 4: Table 2: Please report individual result of prevalence when only one or 2 studies are analysed.
Response 4: Table 2 has been revised accordingly adding results of even one study. Thank you.

Point 5: Fig 3: Pooled estimate is not relevant as there are differences by species and regions..etc. as shown in subgroup analyses. Please sort by %, not alphabetically.
Response 5: The authors understand the reviewer's concern on this point, however the Meta analysis concept is based mostly on pooling findings together. The pooled estimate is for IPPs and we have defined IPPs in method section as detection of one or more of the following intestinal parasites: E. histolytica/dispar, Giardia spp., Cryptosporidium spp., E. coli and other non-pathogenic protozoan parasites. The address the heterogeneity seen from this figure; we further assessed the estimation through subgroup analyses. We hope that it's understandable now. In addition, the all the forest plot figures have been sorted out based on the prevalence results in addition to adding the weight of the studies. We hope it's alright now. Thank you.

Point 6: Fig 4: it is weird that most of the points are outside the funnel and only 3 outliers were identified by the Galbraith plot.
Response 6: Funnel plot results and Galbraith plot results do not depict the same. Funnel plot represents publication bias and studies outside the 95% CI border does not represent those studies to be an outlier. Rather you can easily correlate the updated forest plot and Galbraith plot where the identical outlier studies are clearly visualised as outliers on the forest plot as well. We hope this clarifies the inquiry. Many thanks for the comment.

Editorial Comments

Editorial and Data Presentation Modifications?

Use this section for editorial suggestions as well as relatively minor modifications of existing data that would enhance clarity. If the only modifications needed are minor and/or editorial, you may wish to recommend “Minor Revision” or “Accept”.

Point 1: Line 48 replace the dot after Protozoan parasites with a comma.
Response 1: Done (Line 47).

Point 2: Line 58 remove "is the first", as all publications should be novel this statement is redundant.
Response 2: Removed (Line 56).

Point 3: Line 59 "To date...burden of IPPs" is redundant, consider removing.
Response 3: Removed.

Point 4: Line 86 General recommendation, valid for the all manuscript, consider indicating Cryptosporidium spp. and not Cryptosporidium parvum, as there are many species of Cryptosporidium that can cause illness in humans.
Response 4: Revised accordingly (Line 86).

Point 5: Line 87 "Infection by...malaria and schistosomiasis (7)" is not a finding from the reference 7, kindly seek the primary source and update in the manuscript.
Response 5: By checking the reference list of the above article we have found that the source of this statement are (Voigt H, et al 1999) and (L'Her P, 2005) which are very old references, we have tried to find recently published references but we couldn’t, therefore the statement has been revised and reference 7 was replaced

Point 6: Line 88 Replace C. parvum with Cryptosporidium spp. as reference 8 indicates that the most common Cryptosporidium species are hominis and parvum.
Response 6: Done.

Point 7: Line 115 Replace estiamte with estimate.
Response 7: Done.

Point 8: Line 128 Is it not supplementary table 2?
Response 8: Revised accordingly.

Point 9: Line 141-149 According to the inclusion and exclusion criteria, the following study https://www.ajtmh.org/view/journals/tpmd/81/5/article-p799.xml would be eligible for the analysis, please provide reasons for its exclusion and update the section accordingly.
Title + abstract
Response 9: In this study, literature search, selection of publications, and reporting of results were conducted according to the PRISMA guidelines. Because the above study does not contain any information about protozoan parasites in the title or abstract, it was not eligible for full-text screening.

Point 10: Line 153 Is it C. parvum or Cryptosporidium spp.? There are other species of Cryptosporidium infecting humans why were the selection restricted to C. parvum?
Response 10: *Cryptosporidium* spp.

Point 11: Line 182 Please indicate what REML stands for.
Response 11: REML stand for Restricted Maximum Likelihood and it has been indicated

Point 12: Line 242 Table 3 and not 2.
Response 12: Done.

Point 13: Review formation on reference 33.
Table 1 line n°16 Dyab 2016 used modified Zielh-Nelseen to identify Cryptosporidium, which could not indicate the specie, please change in the reported parasite to Cryptosporidium spp.
Response 13: Revised accordingly.

Point 14: Line 191-192 "moderate-quality studies (high risk of bias)" should it not be low-quality studies (high risk of bias) as indicated in line 175, please recheck which studies were excluded and update the analysis if required.
Response 14: The authors sincerely apologise for the typo; the statement has been revised**.**

Point 15: Line 282-283 Why were the studies that uses non-microscopic detection method excluded? Aren't those the most sensitive?
Response 15: Yes, we agree that those are the most sensitive, which is why they were excluded during the sensitivity analysis to ensure the robustness of our findings.

Point 16: Line 285 Were low-and-moderate quality studies removed on the sensitivity analysis or the moderate quality studies?
Response 16: Moderate quality studies were removed during the sensitivity analysis the paragraph was revised accordingly.
